# An Uncommon Coexistence of Ischemic Stroke in a Multiple Sclerosis Patient With Duplicated Superior Cerebellar Artery: A Case Report From Saudi Arabia

**DOI:** 10.7759/cureus.60218

**Published:** 2024-05-13

**Authors:** Bashaier G AlQahtani, Naim Kajtazi, Hanan K Aljaidi, Khawlah Alhatlany

**Affiliations:** 1 Neurology Department, Prince Sultan Military Medical City, Riyadh, SAU

**Keywords:** saudi arabia, young man, superior cerebellar artery, multiple sclerosis, stroke

## Abstract

The case describes a middle-aged man with well-managed multiple sclerosis who experienced an acute ischemic stroke attributed to a rare anatomical variant - a duplicated left superior cerebellar artery (SCA). Despite his prior lack of neurological issues, the patient presented with severe headaches and walking difficulties. Imaging confirmed the ischemic stroke in the left SCA territory and revealed the duplicated left SCA. This case highlights the importance of considering cerebrovascular anatomy in stroke risk assessment, especially in individuals with unique vascular features such as duplicated SCAs, which can predispose to vascular complications.

## Introduction

The superior cerebellar artery (SCA) typically arises from the basilar artery, situated below the posterior cerebral artery (PCA). It primarily supplies the upper cerebellum, dentate nucleus, a significant portion of cerebellar white matter, specific regions of the midbrain, and the superior and middle cerebellar peduncles [1].

Although infrequent in occurrence and rarely found missing, duplication of the SCA is relatively common. Unilateral duplication is observed in approximately 28% of cases, bilateral duplication in around 8% of cases, and triplication in around 2% of cases. In instances of duplication, the upper branch commonly stems from the PCA, providing blood to the vermis, while the lower branch supplies the cerebellar hemisphere [2,3].

This study presents the first-ever neuroradiological and neuropathological visualization of a duplicated origin of the SCA, which is associated with multiple sclerosis (MS) and an ischemic stroke affecting the corresponding cerebellar area.

## Case presentation

The patient, a 40-year-old male, was previously diagnosed with MS at the age of 35. He has been under effective management with fingolimod, showing no neurological deficits attributable to MS prior to this event. The patient presented to the emergency department with a sudden onset of severe occipital headache, unsteady gait, nausea, and vomiting. These symptoms were new and had not been previously reported by the patient.

Upon admission, the patient's vital signs were noted, with a blood pressure of 160/80 mmHg, indicative of a hypertensive response possibly related to acute distress or pain. Neurological assessment using the National Institutes of Health Stroke Scale (NIHSS) yielded a score of 2, reflecting mild impairment. The deficits noted included left-sided ataxia and an ataxic gait, suggesting cerebellar involvement.

Initial laboratory tests, including a complete blood count, electrolytes, renal function tests, and coagulation profile, returned within normal limits and showed no immediate abnormalities that could explain the acute symptoms.

**Figure 1:**
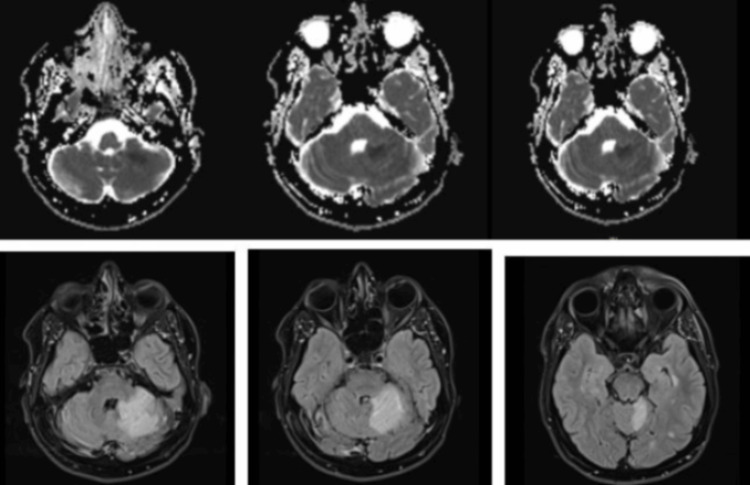
MRI of the brain showing the existence of an acute ischemic stroke at different levels.

A brain magnetic resonance imaging (MRI) was promptly performed, revealing acute ischemic changes in the territory of the left SCA (Figure 1). Notably, there were no new demyelinating lesions that would suggest an exacerbation of the patient's underlying MS.

**Figure 2:**
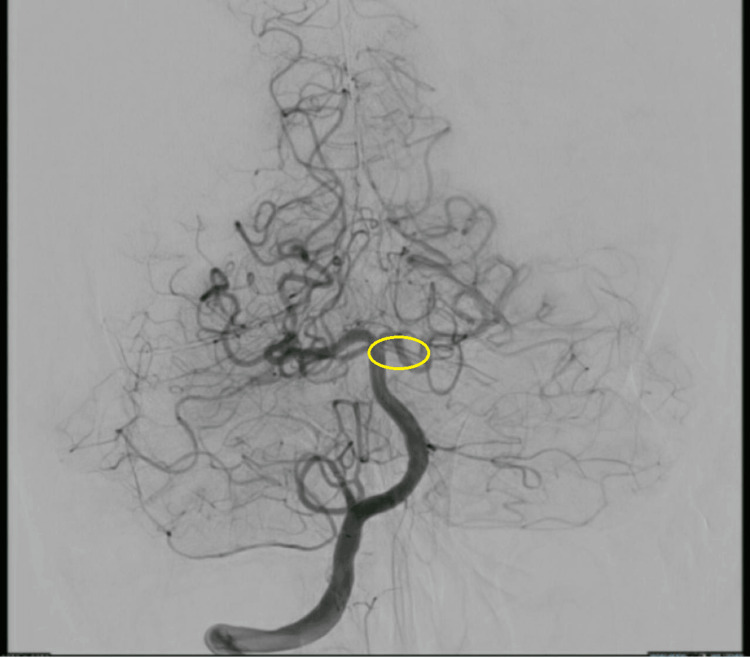
Cerebral angiography showing a duplicated superior cerebellar artery (yellow circle).

Further vascular imaging was conducted using computed tomography angiography (CTA), which showed inconclusive results concerning the presence of a thrombus or arterial dissection in the right SCA (Figure 2). To resolve these uncertainties, a cerebral angiography was undertaken, which confirmed a duplicated left SCA, a rare anatomical variant that might predispose to vascular complications such as stroke.

Given the acute presentation and imaging findings suggestive of stroke, a comprehensive stroke workup was initiated. This included echocardiography and transesophageal echocardiography to exclude a cardiac source of emboli and Holter monitoring to detect any paroxysmal atrial fibrillation that could contribute to embolic stroke. Tests for vasculitis and a hypercoagulable state were also performed to rule out other less common causes of stroke in younger patients. All these investigations returned negative, effectively ruling out secondary causes for the ischemic event.

This case underscores the complexity of diagnosing acute ischemic stroke (AIS) in patients with pre-existing neurological conditions such as MS. The presence of a duplicated SCA in this patient highlights the need for careful vascular imaging in cases of unexplained neurological symptoms, as anatomical variations can significantly influence stroke risk and therapeutic approaches. The management of such cases requires a multidisciplinary approach to effectively address both the acute event and the underlying chronic disease.

The patient provided verbal informed consent for the publication of this case report. He was made aware of the intent to publish the details of his medical case and agreed to have it shared for educational and scientific purposes, while understanding that all identifying information would be removed to protect his privacy.

## Discussion

The case under consideration illuminates the diagnostic complexities and therapeutic challenges encountered when addressing ischemic stroke precipitated by atypical vascular anomalies in individuals with MS. The patient, a 40-year-old male, exhibited acute neurological symptoms, including severe occipital headaches and cerebellar ataxia. This clinical scenario underscores the imperative to contemplate vascular anomalies as potential etiologies for cerebrovascular events in MS patients, particularly those undergoing disease-modifying therapies.

Upon comparative analysis with extant literature, our case resonates with prior reports in terms of clinical manifestations yet distinguishes itself through its unique diagnostic features. The patient underwent an extensive diagnostic evaluation to exclude an MS flare-up, ultimately leading to the identification of ischemic stroke attributable to a duplicated SCA, with one branch supplied by a persistent trigeminal artery variant ascertained via magnetic resonance angiography [4].

The therapeutic regimen, which encompassed antiplatelet therapy in conjunction with disease-modifying agents, proved efficacious in ameliorating symptoms and enhancing the patient's quality of life. Nonetheless, the management of long-term disease-modifying therapy presented a conundrum, balancing potential risks against therapeutic benefits, thereby necessitating vigilant monitoring and consistent follow-up.

Anatomical analysis revealed that 11.7% of patients exhibited typical cerebellar artery anatomy. Vascular anomalies were noted, including the continuation of the vertebral artery as the posterior inferior cerebellar artery (PICA) in 4.4% of cases. Conversely, the absence of the anterior inferior cerebellar artery (AICA) was observed in 36.1% of individuals, and PICA was absent in 6.7% of cases. Additional vascular irregularities included an extradural origin of PICA in 20.8% of patients, duplication of AICA in 7.9%, duplication of SCA in 20.5%, and early bifurcation of SCA in 9.4%. A common trunk between SCA and the PCA was present in 9.4% of cases, and in 8.2% of cases, the SCA originated from the PCA [2].

A comprehensive systematic review encompassing studies from 1983 to 2017 involving more than 380,000 MS patients elucidated an elevated stroke risk within this cohort [4-6], particularly ischemic stroke, when juxtaposed with the general populace [6]. The incidence of stroke in MS patients was 3.48-fold higher at the one-year mark and 2.45-fold higher over 10-13 years. The probability of stroke in MS patients was double that of veterans and nearly triple that of the general population [7]. The incidence of ischemic stroke was notably higher in the MS cohort, being sixfold greater than in non-MS individuals [5]. The findings advocate for early preventative measures and treatments to optimize patient prognoses [7].

In the context of AIS, research indicates that an abrupt, substantial influx of lymphocytes into the ischemic brain region instigates localized inflammatory responses, exacerbating tissue necrosis and clinical outcomes. A preliminary clinical trial demonstrated that oral fingolimod administration within 72 hours post-AIS attenuated lymphocyte migration to the brain. This intervention significantly curtailed the progression of secondary lesions, diminished microvascular permeability, and fostered improved clinical outcomes during the acute phase and at the three-month follow-up [5].

## Conclusions

In this case report, we aim to highlight the importance of recognizing a duplicated SCA when diagnosing patients with MS. Understanding how blood flow variations in the brain can influence stroke risk is crucial for these patients. It is essential to conduct further research to enhance our knowledge of the pathophysiological mechanisms behind strokes caused by this arterial duplication. Such insights will aid in refining management strategies using appropriate disease-modifying therapies, ultimately improving treatment outcomes for patients with similar conditions.
